# Robotic management of complex ileal conduit complications

**DOI:** 10.1007/s11701-026-03565-5

**Published:** 2026-06-19

**Authors:** Juan P. Dugarte, Federico Eskenazi, Ignacio A. Bianco, Jennifer Lu, Rodrigo Guevara, Luis G. Medina, Rene J. Sotelo

**Affiliations:** 1 Urology, University of Southern California, Los Angeles, USA; 2 University of Bologna, Bologna, Italy; 3 Urology, Medical University of South Carolina, Charleston, USA

**Keywords:** Ileal conduit, Urinary diversion, Ureteroenteric stricture, Uretero-ileal anastomosis, Robotic reconstruction, Ureteralreimplantation, Conduit torsion, Parastomal hernia, Crossed ureters, Reconstructive urology

## Abstract

Ileal conduit urinary diversion is associated with complications involving the uretero-ileal anastomosis, conduit segment, stoma, and abdominal wall. Complex complications may require individualized reconstructive strategies based on the specific anatomic failure mechanism. A video-based descriptive technical analysis was performed using a narrated operative recording of four robot-assisted reconstructive procedures for ileal conduit complications: right uretero-ileal stricture, bilateral uretero-ileal stricture, delayed conduit torsion with parastomal hernia, and crossed ureters after ileal conduit diversion. The unilateral stricture repair used antegrade methylene blue injection, needle confirmation of the ureteral lumen, ureteral incision and spatulation, ileal conduit incision, stenting, and running side-to-side uretero-ileal reimplantation. The bilateral stricture repair required mobilization and removal of the prior conduit, creation of a longer ileal conduit, bowel reconstruction, ICG-assisted ureteral identification, bilateral ureteral spatulation, stenting, and running uretero-ileal reimplantation. The torsion case involved circumferential stomal access, fascial opening, detorsion of a 360-degree conduit twist, and parastomal reinforcement with polypropylene mesh. The crossed-ureter revision used bilateral excision of prior anastomoses to healthy tissue, side-specific dye/saline confirmation, bilateral spatulation, double-barrel ureteral reconstruction, and circumferential anastomosis to the conduit. Complex ileal conduit revision can be organized around problem recognition, ureteral identity confirmation, preparation of healthy tissue, tension-aware reconstruction, and repair selection based on the underlying anatomic defect. This mechanism-based approach provides a structured framework for robot-assisted correction of uretero-ileal strictures, conduit torsion, and ureteral malorientation after ileal conduit diversion.

**Supplementary Information:**

The online version contains supplementary material available at 10.1007/s11701-026-03565-5.

## Introduction

Ileal conduit diversion remains a widely used urinary diversion after radical cystectomy. Successful robot-assisted ileal conduit construction requires careful bowel isolation, ureteroenteric anastomotic planning, conduit maturation, and preservation of orientation throughout reconstruction [1]. The choice between intracorporeal and extracorporeal diversion after robot-assisted radical cystectomy is associated with distinct perioperative and complication profiles [2]. Reconstructive correction after conduit-related complications, however, represents a separate technical challenge from primary diversion.

Ileal conduit complications may involve the ureteroenteric anastomosis, conduit segment, stoma, abdominal wall, or upper urinary tract [3,4]. Ureteroenteric stricture is especially important because it may compromise upper-tract drainage and may require endoscopic, percutaneous, or reconstructive management depending on anatomy, tissue quality, and severity [3,5,6]. Stomal and conduit-related complications, including parastomal problems and rare conduit torsion or volvulus, require a different operative strategy because the underlying issue may be mechanical rather than purely anastomotic [4,7].

Robot-assisted correction of complex ileal conduit complications can be organized into four reconstructive scenarios: unilateral uretero-ileal stricture, bilateral uretero-ileal stricture requiring new conduit creation, delayed conduit torsion with parastomal hernia, and crossed ureters requiring corrective reconstruction.

## Methods

The source material consisted of a narrated operative recording of robot-assisted management of complex ileal conduit complications. The analysis was limited to operative details directly visible in the recording, directly stated in the narration, written in captions or slides, or supported by embedded references.

Patient-level characteristics were summarized to contextualize the anatomic failure pattern, clinical presentation, preoperative renal status, indication for robotic revision, and postoperative course for each case (Table 1).

**Table 1:** Patient-level case characteristics

Variable	Case 1	Case 2	Case 3	Case 4
Age, sex	72, male	67, male	69, male	64, male
Original indication for ileal conduit	MIBC	MIBC	MIBC	MIBC
Time from ileal conduit to revision	4 months	3 months	6 months	7 months
Presenting symptoms	Pelvic pain	Lumbar pain and recurrent UTIs	Urinary retention and persistent UTIs	Asymptomatic
Preoperative imaging findings	Right hydronephrosis	Bilateral hydronephrosis	NR	Stable mild bilateral hydronephrosis with delayed right nephrogram
Nephrostomy status	None	Bilateral nephrostomies	None	Bilateral nephrostomies
Preoperative renal function	Cr 1.72 mg/dL	Cr 2.64 mg/dL	Cr 1.40 mg/dL	Cr 1.30 mg/dL
Prior interventions	None	Bilateral nephrostomy placement	None	Bilateral nephrostomy placement
Indication for robotic revision	Pain with right hydronephrosis	Rising creatinine with bilateral obstruction	Urinary retention within the ileal reservoir	Nephrostomy-dependent bilateral hydronephrosis with delayed right nephrogram
Follow-up duration	1 year	1 year	2 years	1 year
Postoperative result	Improved pain, hydronephrosis, and creatinine	Creatinine improved to 1.45 mg/dL	Adequate urine flow through the ileal segment	Stable bilateral hydronephrosis; split renal function left 64.7%, right 35.3%

Abbreviations: Cr, creatinine; MIBC, muscle-invasive bladder cancer; NR, not reported; UTI, urinary tract infection

The cases were selected from available operative recordings demonstrating distinct anatomic mechanisms of complex ileal conduit failure. They were not selected according to postoperative outcome. All procedures were performed by the same surgeon, allowing consistent assessment of operative strategy, reconstructive decision-making, and technical sequence across cases.

### Surgical technique

#### General operative concept

The operation is organized around the specific anatomic failure mechanism. The shared reconstructive principles are exposure of the conduit and ureteral segment, confirmation of ureteral identity or lumen when necessary, excision or bypass of compromised tissue, preparation of healthy ureteral ends, stented reconstruction when indicated, and selection of an anastomotic configuration that fits the available anatomy.

The patient is placed in a modified lithotomy position with Trendelenburg. The Da Vinci Xi surgical system must be docked from the patient’s side. Access to the abdomen is performed transperitoneally with an open technique (Hasson’s technique), usually with a 6-port configuration. Ports were placed around a right para-umbilical ileostomy: 12-mm AirSeal and 5-mm assistant ports superiorly, with 8-mm camera, 8-mm right, 8–12-mm left, and 8-mm fourth-arm robotic ports arranged to preserve spacing and triangulation.

#### Right uretero-ileal stricture

The first scenario involves robot-assisted correction of a right uretero-ileal stricture. Methylene blue is instilled through a nephrostomy tube to assist ureteral localization. The ureteral lumen is then confirmed with a needle before incision. This sequence is particularly relevant in reoperative surgery, where fibrosis and altered tissue planes may make the ureter difficult to distinguish from surrounding scar.

After confirmation, the ureter is incised proximal to the stricture and spatulated. An incision is made in the ileal conduit. A running side-to-side uretero-ileal anastomosis is initiated using Quill suture. A ureteral stent is inserted, and the reimplantation is completed.

The technical priority is controlled identification before reconstruction. Ureteroenteric stricture management depends on the location and length of narrowing, tissue quality, and ability to restore unobstructed drainage [3,5]. Endourologic and stent-based strategies may be appropriate for selected benign upper-tract complications after urinary diversion, whereas complex or unfavorable strictures may require reconstructive correction [5,6].

#### Bilateral uretero-ileal stricture

The second scenario involves bilateral obstruction with proximal ureteral stenosis. The reconstructive strategy is creation of a new, longer ileal conduit with bilateral ureteral reimplantation.

The existing ileal conduit is identified and mobilized. The bowel segment is divided with a stapler, and the prior conduit is removed. A new ileal conduit is fashioned with adequate length for bilateral ureteral reimplantation and stoma creation. The isolated ileal segment is measured using a 10-cm suture reference. Bowel continuity is restored with an enteroenteric anastomosis, with attention to tension-free alignment and an intact hemostatic staple line.

The left ureter is identified using ICG injected through nephrostomy access. The ureter is spatulated, a stent is inserted, and the anastomosis is completed using running 4 − 0 Monocryl in a side-to-side fashion. The same sequence is then performed for the right-sided stricture.

Bilateral reconstruction requires attention to conduit length, ureteral reach, orientation, vascular preservation, and avoidance of tension. Bowel-segment planning, ureteral orientation, and ureteroenteric anastomotic construction are central to successful robot-assisted conduit creation and become even more important during reoperative reconstruction [1]. Prior surgery and fibrosis may reduce mobility and increase the risk of tension or compromised tissue.

### Delayed ileal conduit torsion with parastomal hernia

The third scenario involves delayed ileal conduit torsion in the setting of a large parastomal hernia. Intraoperative assessment identifies a 360-degree conduit torsion. A circumferential incision is made approximately 1 cm from the stoma edge, and the fascia is opened to allow visualization and detorsion of the conduit.

After detorsion, a nonabsorbable polypropylene mesh with a central opening is placed around the advanced conduit and secured to the anterior abdominal wall using a mesh tacker. The repair addresses both the twisted conduit and the associated stomal-abdominal wall defect.

This complication differs from ureteroenteric stricture because the dominant problem is mechanical distortion of the conduit-stoma axis. Ileal conduit volvulus is a rare but recognized cause of conduit dysfunction after urinary diversion [7]. Stomal and abdominal wall complications are important contributors to late morbidity after conduit urinary diversion [4]. Revision, therefore requires correction of conduit orientation as well as reinforcement of the surrounding abdominal wall when a parastomal defect is present.

### Crossed ureters after ileal conduit diversion

The fourth scenario involves corrective surgery for crossed ureters after ileal conduit diversion. The ureters are crossed before their anastomosis to the ileal segment. Neither ureter appears obstructed during the initial intraoperative assessment. Four months later, CT imaging demonstrates mild bilateral hydroureteronephrosis, bilateral ureteral twisting, and right ureteral stenosis at the ileoureteric junction.

The prior uretero-ileal anastomoses are excised to reach healthy ureteral tissue bilaterally. Methylene blue is injected through the right nephrostomy tube, and saline is injected through the left nephrostomy route to confirm ureteral identity. Both ureters are trimmed proximally to healthy tissue and spatulated.

Because the ureters are close together, a double-barrel, or “pants,” configuration is selected to preserve vascular supply. The ureters are first joined mucosa-to-mucosa using 5 − 0 Monocryl. A circumferential anastomosis to the ileal conduit is then performed using 3 − 0 Quill suture at the revised anastomotic site.

The key technical problem is reliable laterality confirmation before reconstruction. Crossed or twisted ureters may create misleading visual orientation. Side-specific injection through nephrostomy access provides a practical method for confirming identity before definitive anastomosis. The double-barrel configuration limits the need for extensive separation of closely approximated ureters and is selected to reduce the risk of devascularization.

Available perioperative and follow-up outcomes were summarized for each case, including operative time, estimated blood loss, conversion, intraoperative complications, length of stay, stent duration, reintervention, and follow-up duration (Table 2).

**Table 2:** Perioperative and follow-up outcomes

Variable	Case 1	Case 2	Case 3	Case 4
Operative time	150 min	210 min	120 min	240 min
Estimated blood loss	50 mL	50 mL	50 mL	100 mL
Conversion to open surgery	No	No	No	No
Intraoperative complications	None	None	None	None
Length of stay	6 days	6 days	4 days	6 days
Stent duration	4 weeks	4 weeks	N/A	4 weeks
Reintervention, recurrence, or failure	None	None	None	None
Follow-up duration	1 year	1 year	2 years	1 year

Abbreviations: EBL, estimated blood loss; N/A, not applicable

## Discussion

Complex ileal conduit complications require a tailored reconstructive strategy because the anatomic failure mechanism differs across cases. Uretero-ileal stricture, bilateral conduit/anastomotic failure, conduit torsion, and crossed ureters are not variations of a single problem; each requires a different operative solution.

The stricture repairs emphasize ureteral localization, lumen confirmation, spatulation, stenting, and reimplantation. These steps are consistent with broader principles in ureteroenteric stricture management, where decision-making depends on stricture anatomy, tissue viability, and the feasibility of restoring upper-tract drainage [3,5,6]. In the unilateral case, reconstruction is focused on the stenotic uretero-ileal junction. In the bilateral case, the problem is more extensive and requires replacement of the conduit itself to create adequate length for bilateral reimplantation.

The torsion repair illustrates a separate category of conduit complication. Mechanical obstruction of the conduit-stoma axis requires restoration of conduit orientation and attention to the fascial or parastomal defect. Although uncommon, ileal conduit volvulus can produce clinically significant conduit obstruction and requires recognition as a mechanical failure of the conduit-stoma axis [7]. Late complications after conduit diversion may also involve the stoma, abdominal wall, and upper urinary tract [4]. In this setting, detorsion alone may be insufficient when the parastomal defect remains uncorrected.

The crossed-ureter repair is primarily an anatomy-confirmation and reconstruction problem. Reoperative urinary diversion may distort normal landmarks, and visual assessment alone may be unreliable when ureters are twisted or crossed. Selective methylene blue and saline injection through nephrostomy access allows side-specific confirmation before reconstruction. The double-barrel configuration is selected because the ureters are closely approximated and preservation of vascular supply is a stated technical priority.

Several principles apply across the four scenarios. First, anatomy should be confirmed before committing to reconstruction. Second, stenotic or compromised tissue should be excised or bypassed until healthier ureteral tissue is available. Third, ureteral spatulation helps create a wider anastomotic opening. Fourth, conduit length and orientation are essential reconstructive variables. Fifth, stomal and abdominal wall mechanics must be addressed when the complication involves torsion or parastomal hernia.

Robot-assisted ileal conduit revision requires careful bowel handling, ureteral orientation, anastomotic planning, and selection of the appropriate reconstruction for the specific failure mechanism [1,3,4]. Comparative effectiveness, recurrence prevention, and long-term durability require dedicated clinical outcome studies.

## Conclusion

Robot-assisted correction of complex ileal conduit complications can be organized according to the specific anatomic failure mechanism. Uretero-ileal stricture repair relies on ureteral identification, lumen confirmation, spatulation, stenting, and reimplantation. Bilateral strictures may require conduit replacement and bilateral reconstruction. Conduit torsion requires correction of the conduit-stoma-abdominal wall axis. Crossed ureters require laterality confirmation and reconstruction that preserves ureteral vascularity. The central principle is structured revision: confirm anatomy, prepare healthy tissue, avoid tension, and tailor the repair to the defect.

## Supplementary Information

Below is the link to the electronic supplementary material.

Supplementary Material 1

## Data Availability

No datasets were generated or analysed during the current study.

## References

[1] Medina LG, Baccaglini W, Hernández A, Rajarubendra N, Winter M, Ashrafi AN, Tafuri A, Cacciamani CE, Sotelo R (2019) Robotic intracorporeal ileal conduit: technical aspects. Arch Españoles de Urología 72(3):299–308 30945657

[2] Tanneru K, Jazayeri SB, Kumar J, Alam MU, Norez D, Nguyen S, Bazargani S, Ganapathi HP, Bandyk M, Marino R, Koochekpour S, Gautam S, Balaji KC, Costa J (2021) Intracorporeal versus extracorporeal urinary diversion following robot-assisted radical cystectomy: a meta-analysis, cumulative analysis, and systematic review. J Robotic Surg 15(3):321–333 10.1007/s11701-020-01174-4 33222043

[3] Narita S, Saito M, Numakura K, Habuchi T (2021) Incidence, etiology, prevention and management of ureteroenteric strictures after robot-assisted radical cystectomy: a review of published evidence and personal experience. Curr Oncol 28(5):4109–4117 34677266 10.3390/curroncol28050348 PMC8534632

[4] Kobayashi K, Coel A, Coelho MP, Medina Perez M, Klumpp M, Tewari SO, Appleton-Figueira T, Pinter DJ, Shapiro D, Jawed M (2021) Complications of ileal conduits after radical cystectomy: interventional radiologic management. Radiographics 41(1):249–267 33306453 10.1148/rg.2021200067

[5] El-Nahas AR, Shokeir AA (2010) Endourological treatment of nonmalignant upper urinary tract complications after urinary diversion. Urology 76(6):1302–1308 20472267 10.1016/j.urology.2010.03.014

[6] Tal R, Bachar GN, Baniel J, Belenky A (2004) External-internal nephro-uretero-ileal stents in patients with an ileal conduit: long-term results. Urology 63(3):438–441 15028433 10.1016/j.urology.2003.09.062

[7] Shamavonian R, Andrawis N (2019) Ileal conduit volvulus: rare complication of urinary diversion. BMJ Case Rep 12:bcr–2018 30696652 10.1136/bcr-2018-227924 PMC6350742

